# Solution-Based Ultra-Sensitive Surface-Enhanced Raman Scattering Detection of the Toxin Bacterial Biomarker Pyocyanin in Biological Fluids Using Sharp-Branched Gold Nanostars

**DOI:** 10.1021/acs.analchem.2c03210

**Published:** 2023-01-24

**Authors:** Supriya Atta, Tuan Vo-Dinh

**Affiliations:** † Fitzpatrick Institute for Photonics, Duke University, Durham, North Carolina 27708, United States; ‡ Department of Biomedical Engineering, Duke University, Durham, North Carolina 27708, United States; § Department of Chemistry, Duke University, Durham, North Carolina 27708, United States

## Abstract

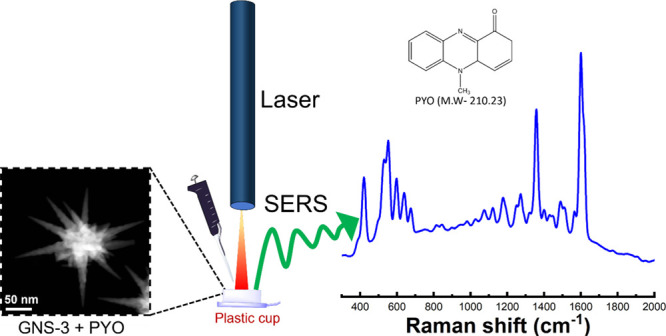

There is a critical need for sensitive rapid point-of-care detection of bacterial infection biomarkers in complex biological fluids with minimal sample preparation, which can improve early-stage diagnosis and prevent several bacterial infections and fatal diseases. A solution-based surface-enhanced Raman scattering (SERS) detection platform has long been sought after for low cost, rapid, and on-site detection of analyte molecules, but current methods still exhibit poor sensitivity. In this study, we have tuned the morphology of the surfactant-free gold nanostars (GNSs) to achieve sharp protruding spikes for maximum SERS enhancement. We have controlled the GNS spike morphologies and optimized SERS performance in the solution phase using para-mercaptobenzoic acid as an SERS probe. To illustrate the potential for point-of-care applications, we have utilized a portable Raman instrument for measurements. For pathogenic agent sensing applications, we demonstrated rapid and sensitive detection of the toxin biomarker pyocyanin (PYO) used as the bacterial biomarker model system. Pyocyanin is a toxic compound produced and secreted by the common water-borne Gram-negative bacterium *Pseudomonas aeruginosa*, a pathogen known for advanced antibiotic resistance and association with serious diseases such as ventilator-associated pneumonia and cystic fibrosis. The limit of detection (LOD) achieved for PYO was 0.05 nM using sharp branched GNSs. Furthermore, as a proof of strategy, this SERS detection of PYO was performed directly in drinking water, human saliva, and human urine without any sample treatment pre-purification, achieving an LOD of 0.05 nM for drinking water and 0.4 nM for human saliva and urine. This work provides a proof-of-principle demonstration for the high sensitivity detection of the bacterial toxin biomarker with minimal sample preparation: the “mix and detect” detection of the GNS platform is simple, robust, and rapid, taking only 1–2 min for each measurement. Overall, our SERS detection platform shows great potential for point-of-need sensing and point-of-care diagnostics in biological fluids.

## Introduction

Rapid and sensitive detection of microbial biomarkers in complex biological fluids with minimal sample preparation is of utmost importance for early-stage diagnosis, which can prevent several bacterial infections and high mortality.^1−3^*Pseudomonas aeruginosa* is one of the most common water-borne Gram-negative pathogens that cause acute and chronic nosocomial infections, and it substantially increases the mortality risk, especially for immunocompromised patients.^4−7^ Therefore, there is an urgent demand to develop a method for rapid detection of *Pseudomonas aeruginosa*.^8,9^ Conventional pathogen detection methods are generally based on the colony culture method, which includes pathogen collection, culturing in multiple media, and performing immunoassay for pathogen metabolites.^10^ It is important to note that a toxin biomarker pyocyanin (PYO) is secreted from *Pseudomonas aeruginosa*. A variety of detection methods such as enzyme-linked immunosorbent assay (ELISA), polymerase chain reaction (PCR), and electrochemical detection methods have been developed for the detection of PYO.^11−14^ Despite their high sensitivity toward the detection of trace amounts of PYO, these methods generally require rigorous sample preparation and pre-purification, and thus, they are elaborate and time-consuming, which restrict their applications for point-of-care onsite detection. Therefore, there is an urgent need for an efficient analytical method for the detection of PYO at the point of need and quick diagnosis.

Surface-enhanced Raman spectroscopy (SERS) is a powerful spectroscopic technique, which provides a vibrational fingerprint for Raman active molecules adsorbed onto a plasmonic nanostructured surface.^15^ Over three decades ago, our laboratory first introduced SERS as an analytical tool for trace analysis^16^ and has developed SERS-active platforms for a wide variety of applications spanning chemical analysis, biological sensing, and medical diagnostic.^17−20^ The SERS approach is suitable for rapid on-site detection because of its simplicity of use and portable feature; furthermore, it could be utilized to directly analyze complex samples such as bodily fluids, human urine, and saliva.^3^ The SERS enhancement is highly dependent on the localized surface plasmon resonance of the metal nanoparticle’s size, shape, and type of metal nanostructure.^15^ The recent growth of nanoparticle synthetic strategies has allowed producing ultrasensitive detection. It is noteworthy that PYO is an SERS-active molecule and there are several previous reports on SERS detection platforms used for PYO detection.^3,21−27^ However, most of them have either involve elaborate and time-consuming sample preparation or exhibit poor sensitivity in the micromolar range. For instance, a biodegradable zein film decorated with gold nanospheres was used for PYO detection in drinking water with an LOD of 25 μM.^28^ Thus far, there is no report on rapid, sensitive, and direct detection of PYO by SERS with minimal sample preparation.^2^ We believe that one of the main challenges is the control and optimization of plasmonics-active nanoparticle morphology that can generate the highest electromagnetic field to achieve sensitive detection.^29^

Among the different sizes and shapes of gold nanoparticles synthesized to date, gold nanostars have attracted great interest in the nanoparticle research community in the last few decades owing to their unique tunable optical properties, excellent biocompatibility, and their application in different fields.^30−37^ Our group first introduced the use of GNS as an SERS-enhancing platform^38^ and subsequently developed the synthesis of biocompatible surfactant-free GNS, and it has been explored in different fields including sensing, imaging, and therapy.^39−41^ Despite significant advances, there is still a need for further investigation to achieve optimum morphology for maximum SERS enhancement. An ideal morphology of GNS should consist of long spikes with sharp tips that can generate intense local electromagnetic fields.^32,42^ As such, the goal of this study was to develop an effective plasmonics-active GNS platform that would provide ultra-high SERS enhancement to directly detect bacterial biomarkers such as PYO in biological fluids rapidly without any pre-purification.

In this study, we have synthesized three different morphologies of GNS (GNS-1, GNS-2, and GNS-3) by changing the concentration of AgNO_3_ from 15 to 120 μM. The SERS measurement studies were performed in the solution phase using a common SERS probe molecule para-mercaptobenzoic acid (MBA) by utilizing a portable Raman instrument. The GNS with maximum spike lengths and sharp spikes (GNS-3) showed the highest intense SERS responses compared to the shorter and less sharp spiked GNS (GNS-1 and GNS-2), and we have achieved 0.1 nM LOD for MBA by using GNS-3. We have explored our SERS detection platform to detect a toxin biomarker PYO by utilizing GNS-3 where we achieved 0.05 nM LOD. Furthermore, this approach was investigated in real-world samples like drinking water, human saliva, and human urine without any sample treatment pre-purification which allows an LOD of 0.05 nM for drinking water and 0.4 nM for human saliva and urine. In this proof-of-principle demonstration study, the ultra-high label-free SERS detection of PYO indicates the great potential of the sharp-branched GNS as a rapid POC testing platform for detection of early-stage bacterial infections which can reduce longer hospital stays, higher medical costs, and increased mortality.

## Results and Discussion

### Synthesis of GNSs

Gold nanostars with three different sizes and morphologies were synthesized followed by our recently reported developed method for surfactant-free GNS synthesis.^30^ It is reported that the Ag^+^ concentration plays a significant role in determining the overall morphology of the resulting GNS including the spike length, spike number, and tip morphology. In general, the spike length is increased with an increased concentration of AgNO_3_. Interestingly, it is reported that the spike does not form without Ag^+^, and the stability of a star-shaped morphology depends on the Ag^+^ concentration.^43^ In this study, we have used three different concentrations of AgNO_3_ (15, 30, and 120 μM) to achieve GNS-1, GNS-2, and GNS-3 morphology.

**Figure 1:**
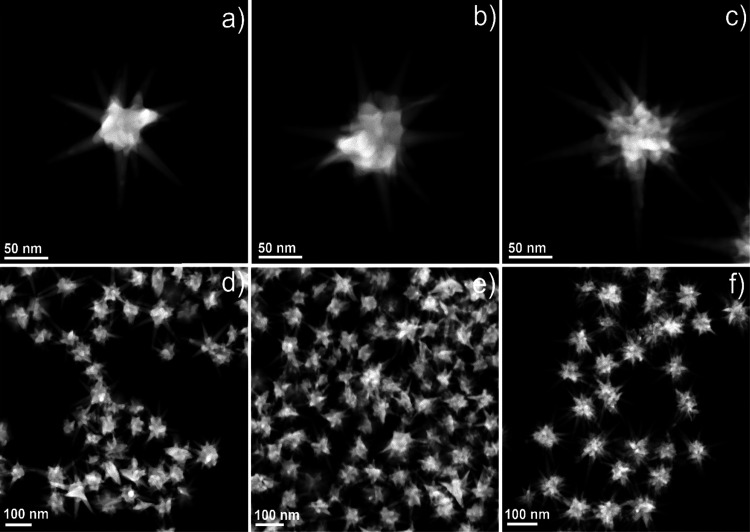
STEM images of GNS-1 (a), GNS-2 (b), and GNS-3 (c) showing that the spike morphology was changed from spherical tips to pointy tips and the spike length was increased from GNS-1 to GNS-3. STEM images with multiple nanostars of GNS-1 (d), GNS-2 (e), and GNS-3 (f) showing high monodispersity of the synthesis.

**Figure 2:**
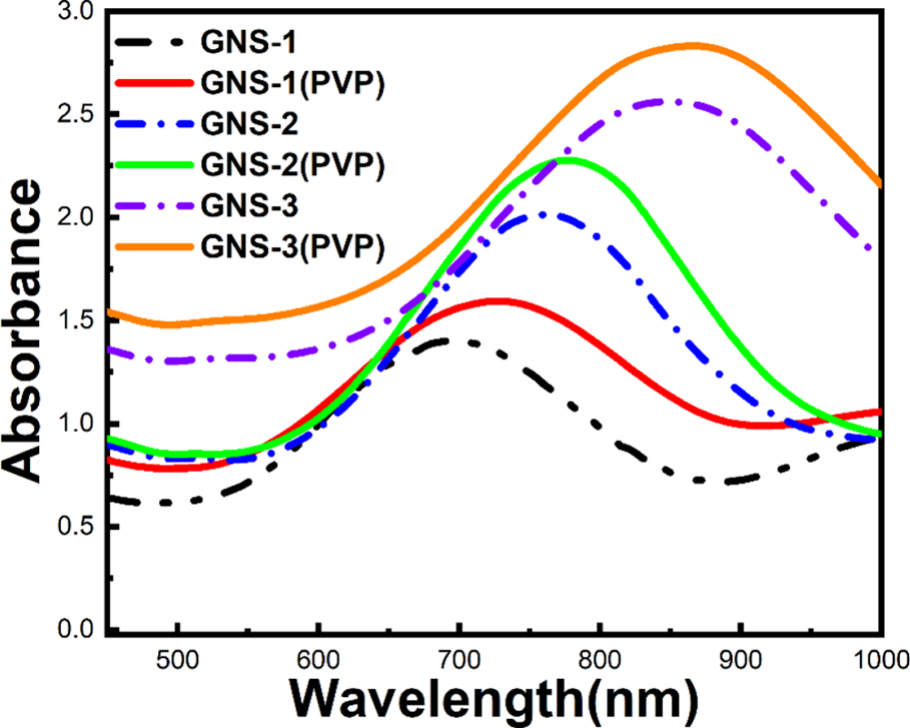
UV–vis spectra of the surfactant-free GNSs (GNS-1, GNS-2, and GNS-3) showing that a red shift from 705 to 850 nm occurs when the GNS morphology changes from GNS-1 to GNS-3 (dotted line) and PVP-coated GNSs which shows that there occurs a 15–20 nm red-shift after PVP coating of the corresponding GNS.

Figure 1 demonstrates representative STEM images of all the GNSs (GNS-1, GNS-2, and GNS-3), which shows that the average spike number and the spike length were increased with increasing AgNO_3_ concentration. The average spike lengths of GNS-1, GNS-2, and GNS-3 were 60 ± 4, 66 ± 5, and 77 ± 6 nm, respectively, where we measured the spike length of 100 spikes for each GNS morphology and the spike length was measured from the surface of the core (the detailed statistical analysis is displayed in Figure S1). It is noteworthy that the tip morphology was changed from less-pointy to sharp-pointy tips when we go from GNS-1 to GNS-3, which became sharper for GNS-3 than for GNS-1 and GNS-2. Figure S2 shows the width of the tips for GNS-1 (8 ± 2 nm), GNS-2 (6 ± 2 nm), and GNS-3 (3 ± 2 nm), where the average width of the spike at the core is around 18 ± 3 nm for all these GNSs. Interestingly, the tip morphology is correlated with the AgNO_3_ concentration. The morphology of the tips is less-pointy in shape at or below 30 μM AgNO_3_ concentration and sharp-pointy in shape at a high concentration of AgNO_3_ (120 μM); this feature could probably be due to the underpotential deposition of Ag which reduces the diffusion of highly energetic gold atoms at the tips toward the more energetically favorable spherical core of the GNS.^43−45^ The optical properties of these GNSs were investigated using UV–vis absorption measurements where the LSPR peak maximum for GNS-1, GNS-2, and GNS-3 are 705, 770, and 850 nm, respectively (Figure 2). The UV–vis spectra of the GNSs show that the LSPR peak was red-shifted from GNS-1 to GNS-3 which is because of spike length increment.^46^ Moreover, we have investigated the batch-to-batch reproducibility of the GNSs. Figure S3 shows the narrow standard deviations of the LSPR peak maximum of ten different batches of GNSs (GNS-1, GNS-2, and GNS-3), indicating high reproducibility of the synthesis.

Surfactant-free GNS has several advantages over the surfactant-based GNSs synthesis such as morphological tunability and biocompatibility.^30,41^ However, the surfactant-free GNSs are not stable in long term, and they tend to be agglomerated after a certain period of synthesis, which is probably due to the instability of highly energetic spikes of GNS.^43^ In order to utilize highly spiked morphology, we have used polyvinylpyrrolidone (PVP, *M*_w_ ∼ 8 k) as a capping agent to reduce the aggregation of GNS. The PVP coating was confirmed by the red-shifting of the LSPR peak of the GNS (Figure 2, solid lines). Moreover, the PVP polymer can help absorb the PYO molecules on the GNS through electrostatic interactions,^47−49^ which might be between the active sites of PYO (N-center) and the carbonyl group of PVP; thereby, the SERS sensitivity can be superior for PVP-capped GNS than surfactant-free GNS.

### SERS Studies

**Figure 3:**
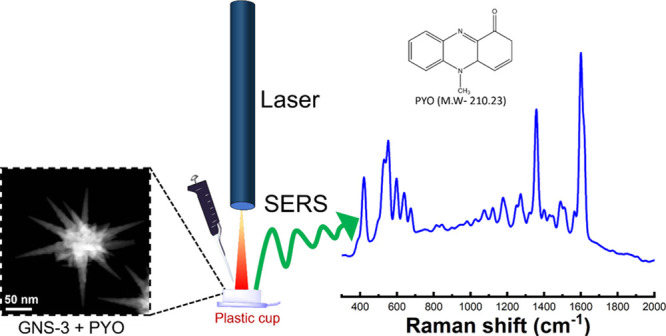
Schematic representation of the solution-based SERS measurement where the analyte (PYO) and SERS-sensor GNS-3 were mixed on a plastic cup cut from a 1.5 mL centrifuge tube where a small piece of aluminum foil was placed at the bottom to prevent the SERS signal interference of the polypropylene plastic cap, and PYO was detected via a portable Raman instrument.

Figure 3 shows a schematic representation of our “mix and detect” detection with the GNS platform. In this study, we have selected five different spots of each sample throughout our SERS measurements. The application of the PVP-capped GNS as a solution-based SERS substrate was first investigated by using MBA as a model analyte molecule. To confirm the sensitivity of the GNSs, we recorded SERS spectra of MBA at 1 μM final concentration. Figure S4 shows the Raman spectra (Figure S4a) and intensity (Figure S4b) at 1078 cm^–1^ of MBA of all the GNSs (GNS-1, GNS-2, and GNS-3) which reveals that GNS-3 has the highest Raman intensity than GNS-1 and GNS-2. GNS-3 exhibits a more intense SERS signal by a factor of ∼2 as compared to the GNS-2 and a factor of ∼3 as compared to the GNS-1. GNS-3 shows the highest SERS enhancement than GNS-1 and GNS-2, which is probably due to the increased surface area provided for molecular adsorption, in addition to the generation of multiple numbers of hotspots, resulting in strong electromagnetic field enhancement localized at the tips of the GNS-3.^30^

**Figure 4:**
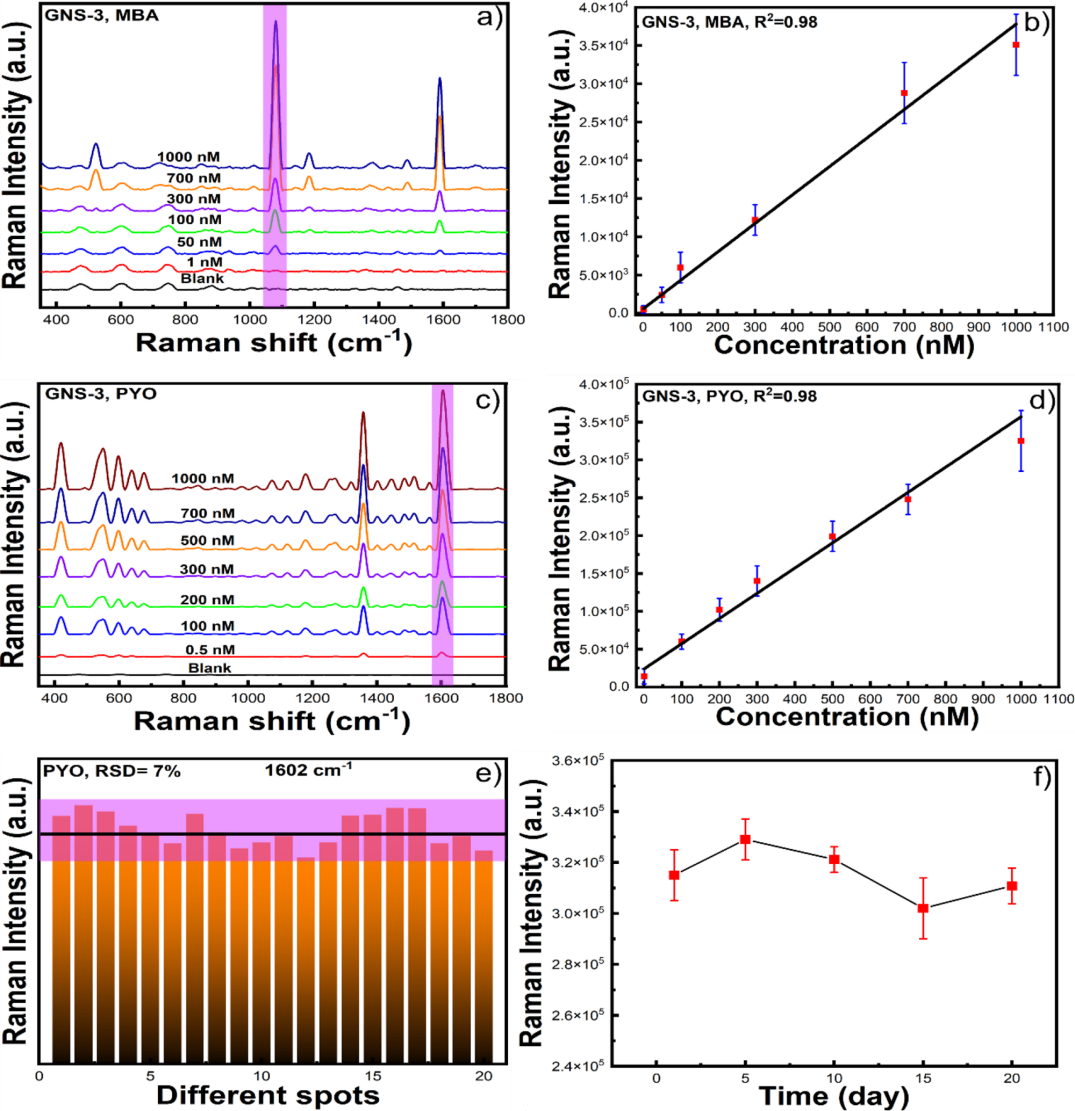
SERS intensity of MBA at 1078 cm^–1^ at different concentrations from 1 μM to 1 nM (a), and the corresponding calibration curve (b) with GNS-3. SERS spectra of PYO at different concentrations (from 1 μM to 0.5 nM) and the blank substrate (c) and the corresponding calibration curve (d) with GNS-3. SERS intensity of PYO at 1602 cm^–1^ for 20 different sample spots (e) and different time intervals (f) which shows that our solution-based SERS substrate is reproducible and stable.

Figure 4a displays the SERS data for concentrations of MBA ranging from 1 μM to 1 nM using GNS-3 as an SERS probe. As seen in Figure 4a, the main characteristic peak intensities of MBA at 1078 and 1590 cm^–1^ increased as the concentration of MBA increased. More importantly, we can observe the peak at 1078 cm^–1^ at 1 nM concentration of MBA. Figure 4b shows the calibration curve where the average peak-height intensity at 1078 cm^–1^ is a function of the concentration of MBA, which shows a linear relationship with the concentration of MBA. From the calibration curve, the limit of detection was estimated to be 0.1 nM, which is determined based on the signal equal to three times the standard deviation of the blank.

Pyocyanin, as mentioned in the Introduction, is a highly toxic chemical released from *Pseudomonas aeruginosa*, which has a life-threatening impact on humans.^6^ After optimizing the performance of the “mix and detect” platform utilizing the sharp spiked GNS-3, we set out to apply the GNS-3 for SERS sensing of PYO. We first compared the SERS enhancement of PYO with GNS-1, GNS-2, and GNS-3. Figure S5 shows that the SERS signal of GNS-3 was more intense by a factor of ∼6 as compared to the GNS-2 and a factor of ∼18 as compared to the GNS-1. The SERS intensity of PYO with GNS-3 was significantly higher than that of the other GNSs which is probably because of the large surface area covered with PVP coating which facilitates to bind with PYO.

**Figure 5:**
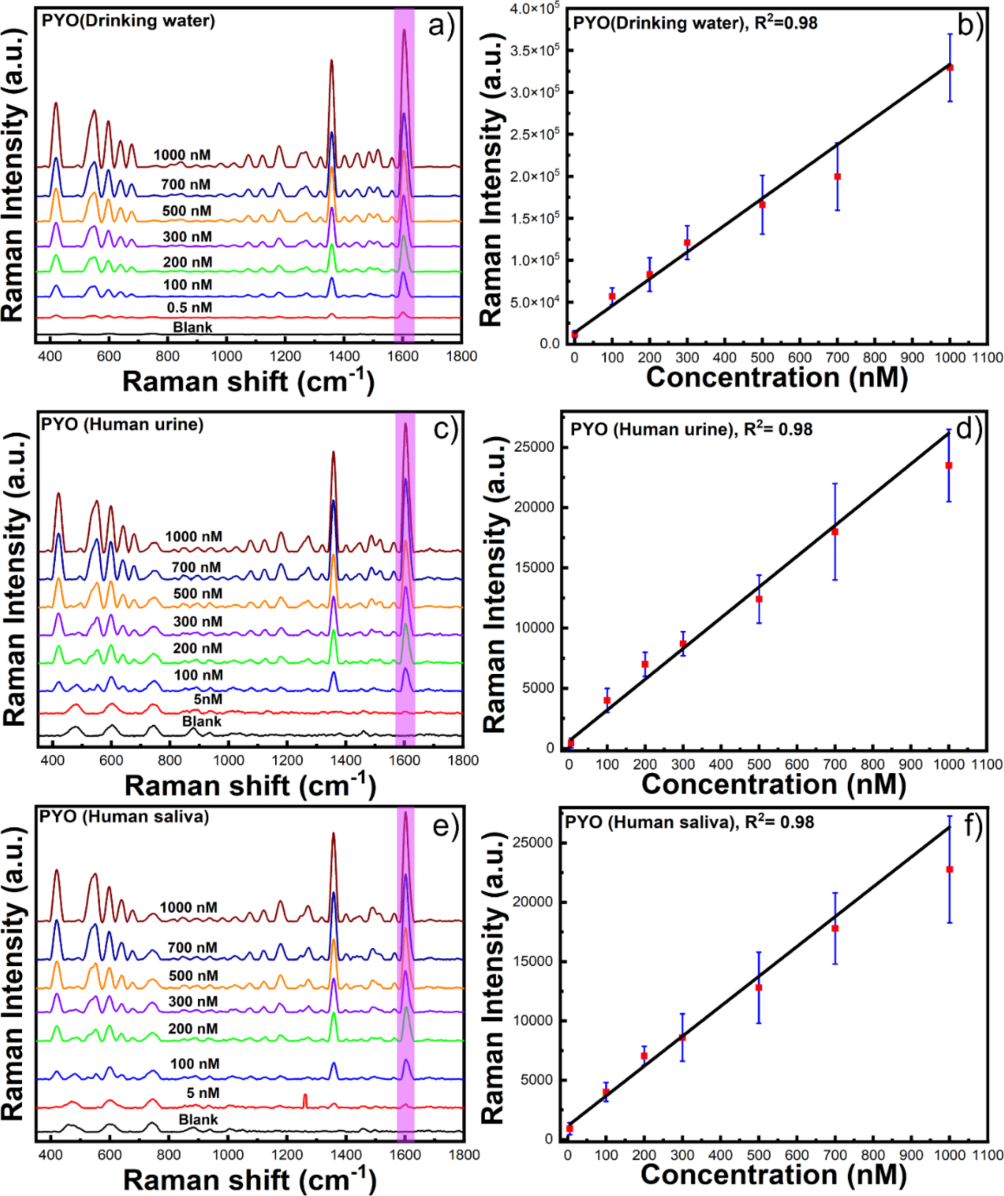
SERS spectrum of PYO in drinking water (a), the corresponding calibration curve for drinking water (b), human urine (c), the corresponding calibration curve for human urine (d), human saliva (e), and the corresponding calibration curve for human saliva (f).

Figure 5a displays the SERS bands of PYO, where the peaks at 417 and 552 can be attributed for the different ring deformations and the peaks at 1357 and 1602 cm^–1^ can be attributed to the aromatic ring stretching,^10^ which are similar to the reported PYO SERS spectra.^2,28^Figure 5b shows the calibration curve of the SERS intensity at 1602 cm^–1^ as a function of the PYO concentration. According to the main characteristic band in the SERS spectrum at 1602 cm^–1^, PYO can be detected to be as low as 0.05 nM. The extremely low LOD of PYO as a solution-based colloidal substrate illustrates the advantages of the GNS-based SERS method over reported methods and would have great potential for rapid, sensitive, on-site detection.^2,3,23^

We have further investigated the reproducibility of our solution-based SERS detection platform which are essential parameters for SERS detection. We first checked the reproducibility by collecting 20 SERS signals from 20 different sample spots of PYO at 1 μM concentration. Figure 4e shows the peak height of the Raman band of PYO at 1602 cm^–1^ of the 20 different sample spots. The relative standard deviation (RSD) for the 1602 cm^–1^ band vibrations of PYO was 7%, which indicates the good reproducibility of our solution-based SERS detection. We have also investigated the batch-to-batch reproducibility of the SERS performance. Figure S6 shows the peak height of the Raman band of PYO at 1602 cm^–1^ of five different batches of GNS-3 with 6% RSD, indicating batch-to-batch reproducibility of GNS-3.

One of the main issues of SERS substrates is their poor stability with time which significantly affects their reproducibility and cost-effectiveness. GNSs are not stable for the long term at room temperature. Therefore, we have stabilized them by using the PVP polymer and stored them in ambient conditions for 20 days. To test the stability of our solution-based SERS detection platform, we measured the SERS spectra of PYO at 1 μM concentration at different storage times for 5 days intervals up to 20 days. Figure 4f shows that there was no significant change observed in the intensity of the selected characteristic peak of the SERS intensity of PYO at 1602 cm^–1^, indicating good SERS stability. Overall, our study demonstrates that the GNS-3 can fulfill the requirements for routine SERS detection with reproducibility and stability.

To investigate the role of PVP, we performed a control experiment where the SERS signal intensity of MBA and PYO was studied with surfactant-free GNS-3 and PVP-capped GNS-3. Figure S7a shows that the SERS intensity of MBA is higher at 1078 cm^–1^ for surfactant-free GNS-3 than PVP-capped GNS-3. Interestingly, the SERS intensity of PYO at 1602 cm^–1^ is higher for PVP-capped GNS-3 than for surfactant-free GNS-3 (Figure S7b). The surfactant-free GNS shows higher SERS intensity for MBA, which might be probably due to close contact of MBA with the nanostar surface, whereas for PVP-capped GNS-3, the PVP polymer prevents close contact of MBA with the nanostar surface.^50^ On the other hand, the SERS signal intensity is higher for PYO with PVP-capped GNS-3, which is probably due to the electrostatic interaction between PVP and PYO.^47−49^ Furthermore, the UV–vis absorbance spectrum after the addition of PYO into PVP-capped GNS-3 (Figure S8) shows that there is no change of the LSPR maximum after PYO addition which indicates that there is no aggregation of GNS after PYO addition and the SERS enhancement is due to close contact of PYO on the PVP layer of GNS.

Moreover, we have investigated the SERS enhancement of PYO with two different molecular weights of PVP (*M*_w_ ∼ 8 and ∼40 k)-coated GNS-3. Figure S9a shows that the SERS enhancement of PYO is almost identical with the molecular weight of PVP, indicating a monolayer or submonolayer of PVP coating on GNS. STEM images of ∼40 k PVP-coated GNS also show that there is no aggregation or a thick layer of PVP coating on GNS (Figure S9b,c).

### SERS Applications

*Pseudomonas aeruginosa* is a waterborne pathogen commonly found in lakes, seawater, drinking water, and even in distilled water.^51,52^ They have ability to colonize with limited available nutrients and they have a high resistance to disinfectants.^53^ They have the capability to form biofilms rapidly through the quorum-sensing system; therefore, they can be easily transmitted to humans and animals through contaminated water.^54−56^ Contact with *Pseudomonas aeruginosa*-contaminated drinking water causes diarrhea and vomiting and they become a serious threat for immunocompromised patients.^57,58^ Additionally, *Pseudomonas aeruginosa* has a characteristic ability to grow in biological fluids and they can be found in saliva and urine.^59^ Therefore, there is a high demand for instant and on-site detection of toxin biomarkers secreted from *Pseudomonas aeruginosa* in drinking water sources.

The goal of this study was to detect the toxin biomarker pyocyanin secreted from *Pseudomonas aeruginosa* in a real-life SERS sensing scenario such as drinking water and human fluids (saliva and urine) without any pre-purification. In this study, the drinking water and human fluids (saliva and urine) were spiked with PYO and used for analysis. We have used our best SERS-performing nanostars, GNS-3, based on the previously discussed promising results. The characteristic band of PYO at 1602 cm^–1^ was clearly detected in the SERS spectra of the real-life samples. Figure 5a,b shows the SERS spectra and the calibration curve of PYO in drinking water where we achieve an LOD of 0.05 nM. Figure 5c–f shows the SERS spectra for different PYO concentrations in human urine and saliva and their corresponding calibration curves. It is noteworthy that the LOD increased to 0.4 nM with human fluids, which is slightly higher than that of the LOD for drinking water. Comparing the SERS signals for the drinking water and human fluids, it was observed that the LOD was higher for human bodily fluids. This could be attributed to the fact that biological molecules in human fluids can partially block the GNS surface and prevent the target analyte from getting adsorbed on or closer to the GNS surface, resulting in a weaker SERS signal. This sensing approach illustrates the advantages of a practical detection technique for bacterial toxins such as pyocyanin in complex environmental samples or biological fluids, where the clinically relevant concentration range for PYO is from 2 to 100 μM,^8^ at the point of need within a short handling period, resulting in outstanding sensing performance.

## Conclusions

This work highlighted the importance of nanoparticle design for the point-of-care SERS platform for rapid sampling and detection of infectious pathogens utilizing sharp-branched GNSs. The effect of GNS morphology on SERS enhancement was investigated with the goal of developing a sensitive SERS platform. We have achieved changes in the morphology of GNS by controlling the concentration of AgNO_3_. We have first optimized the SERS platform for a model analyte MBA where we achieved 0.1 nM LOD. Thereafter, we investigated the detection of the toxin biomarker PYO, and we achieved an LOD of 0.05 nM using sharp branched GNS (GNS-3). Moreover, we have employed this SERS detection platform to detect PYO in drinking water, human saliva, and urine samples without any pre-purification, where we achieved the detection limits of 0.05 nM for drinking water and 0.4 nM for human saliva and urine. The result of this study demonstrates the possibility for rapid and highly sensitive detection of a bacterial toxin biomarker with minimal sample preparation and great potential for point-of-need sensing and point-of-care diagnostics. This study is aimed at providing proof of principle demonstration of the potential for our solution-based SERS detection scheme to detect toxin biomarkers with high sensitivity. Further studies involving clinical samples from patients and healthy individuals will be needed to validate the technique for medical diagnostics at point-of-need.

## Supporting Information Available

Experimental section; statistical distribution of the spike length of the GNSs (Figure S1); width of the tips for GNSs (Figure S2); LSPR peak maximum of GNSs (Figure S3); SERS spectra and SERS intensity of MBA and PYO with GNSs (Figures S4 and S5); SERS reproducibility of GNS-3 (Figure S6); SERS spectra with PVP-capped GNS-3 and surfactant-free GNS-3 (Figure S7); absorbance spectra of PVP-capped GNS-3 before and after PYO addition (Figure S8); SERS spectra of PYO and STEM images of GNS-3 coated with PVP (*M*_w_-8, and 40 k) (Figure S9); and SERS spectra without background subtraction (Figure S10) (PDF)
