# Supplementary material for: Solution-Based Ultra-Sensitive Surface-Enhanced Raman Scattering Detection of the Toxin Bacterial Biomarker Pyocyanin in Biological Fluids Using Sharp-Branched Gold Nanostars
Source: Anal Chem. 2023 Jan 24;95(5):2690–7. doi: 10.1021/acs.analchem.2c03210 (PMC9909734; doi:10.1021/acs.analchem.2c03210)
Supplement: Supplementary file 1 — ac2c03210_si_001.pdf [file ac2c03210_si_001.pdf]

Supporting Information

Solution-based Ultra-Sensitive SERS Detection of the Toxin Bacterial Biomarker Pyocyanin in Biological Fluids Using Sharp-Branched Gold Nanostars

Supriya Atta, [a, b] *Tuan Vo-Dinh* [a, b, c *]

[a] Fitzpatrick Institute for Photonics, [b] Department of Biomedical Engineering, [c] Department of Chemistry, Duke University, Durham, NC 27708, USA

(*) Corresponding Author: tuan.vodinh@duke.edu

EXPERIMENTAL SECTION

Materials and Characterization

Chloroauric acid ($HAuCl_4$), L-ascorbic acid, silver nitrate ($AgNO_3$, 99.8%), pyocyanin, MBA, hydrochloric acid (HCl), trisodium citrate ($Na_3C_6H_5O_7$), and PVP (40k) were purchased from Sigma-Aldrich. PVP (8k) was purchased from Thermo Fisher Scientific. Pooled human urine and saliva were purchased from Innovative Research. The STEM images of the nanostars were collected from Aberration Corrected STEM-Thermo Fisher Titan 80-300. The UV-vis spectra of

the nanostars were recorded using a Shimadzu UV-3600i spectrometer with cuvettes of 1 cm path length.

Synthesis of GNS-1, GNS-2, and GNS-3

Surfactant-free GNS (GNS-1, GNS-2, and GNS-3) were synthesized by following previously reported method.[1, 2] Briefly, 12-15 nm gold nanospheres were synthesized by following a reported procedure.[2] The gold nanospheres were concentrated to 30 times from synthesis concentration and used further for GNS synthesis. For GNS synthesis, 200 μL of 1 N HCl was added to a solution containing 50 mL of 0.5 mM $HAuCl_4$ and 15 μL of the gold nanospheres solution. Then, a certain amount of 3 mM $AgNO_3$ and 1 mL of 100 mM ascorbic acid were added. To achieve GNS-1, GNS-2, and GNS-3, the final concentration of $AgNO_3$ were 15, 30, and 240 μM. The solution was stirred for 2 minutes. Then, 1 mL 1 mM PVP (M_w-8k) solution was added to GNS solution and stirred for 6 hours at room temperature. The solution was centrifuged at 4000 g for 12 min and dispersed in 12.5 mL of Milli-Q water so that the concentration of GNS was four times higher than the synthesized nanostars. The nanostars were stored at room temperature.

Raman Measurements

The SERS measurement was carried out using a lab built portable system having 785 nm laser source (Rigaku Xantus TM-1 handheld Raman device), a fiber optic probe (InPhotonics RamanProbe), a spectrometer (Princeton Instruments Acton LS 785), and a CCD camera (Princeton Instruments PIXIS: 100BR_eXcelon). Laser power of the Rigaku Xantus TM-1 was set at 200 mW and the CCD camera exposure time was set at 2 s. The SERS measurement was standardized using ethanol. The SERS measurement involves a minimal sample preparation in which 300 μL of the gold nanoparticle solution (10 μL stock solution of GNS + 287 μL of Milli-Q-water) were thoroughly mixed with 3μL of analyte solution in a plastic cup cut from a 1.5 mL

centrifuge tube which was covered with aluminum foil to prevent signal interference from the polypropylene well plate.

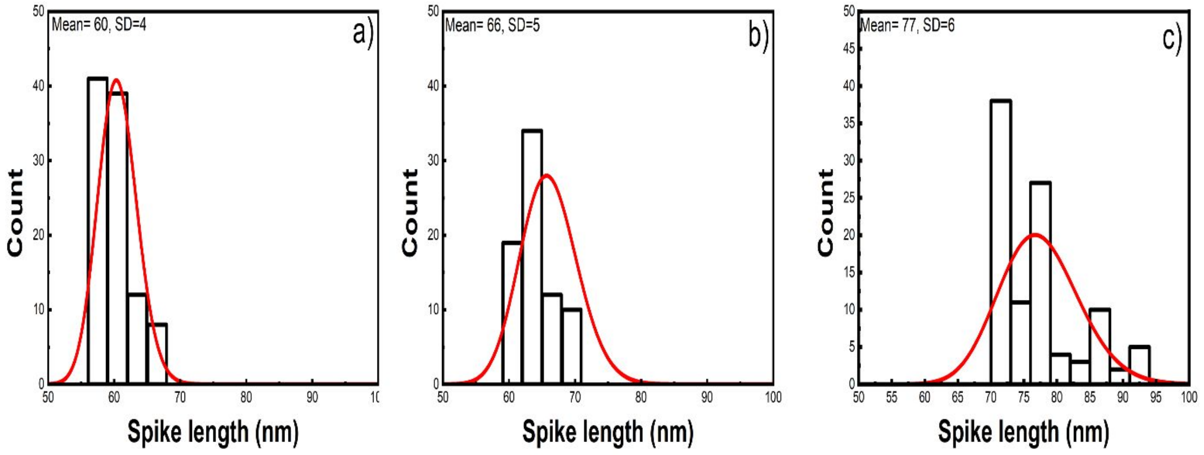

Figure S1. Statistical distribution of the spike length of the GNSs (a) GNS-1, (b) GNS-2, (c) GNS-3) where the distance measured from the core to the tip.

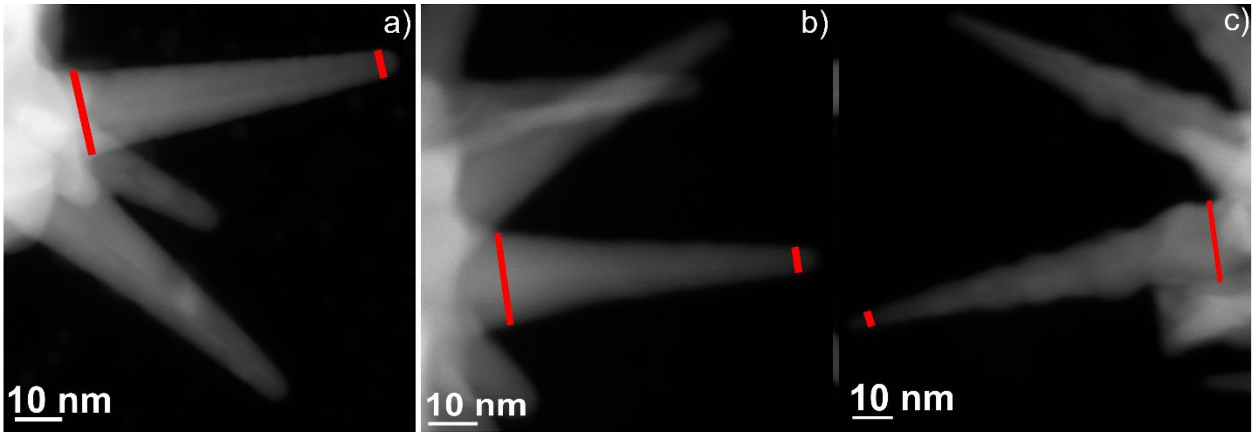

Figure S2. The width of the tips for GNS-1 (8±2 nm), GNS-2 (6±2 nm), and GNS-3 (3±2 nm), where the width of the spike at the core is around 18 nm for all these GNSs.

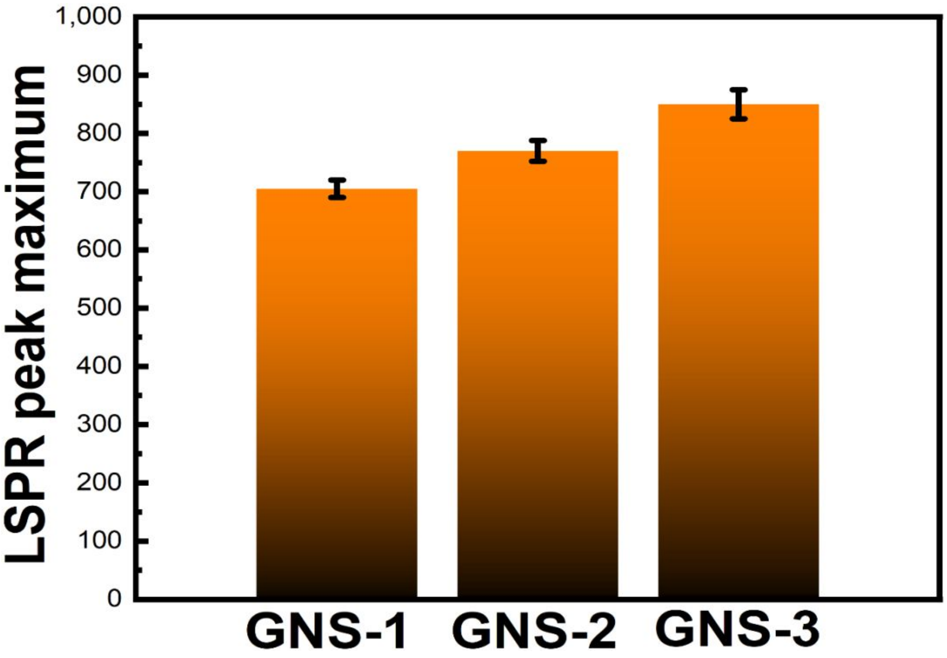

Figure S3. Evolution of the LSPR peak maximum of ten different batches of PVP capped GNS-1, GNS-2, and GNS-3.

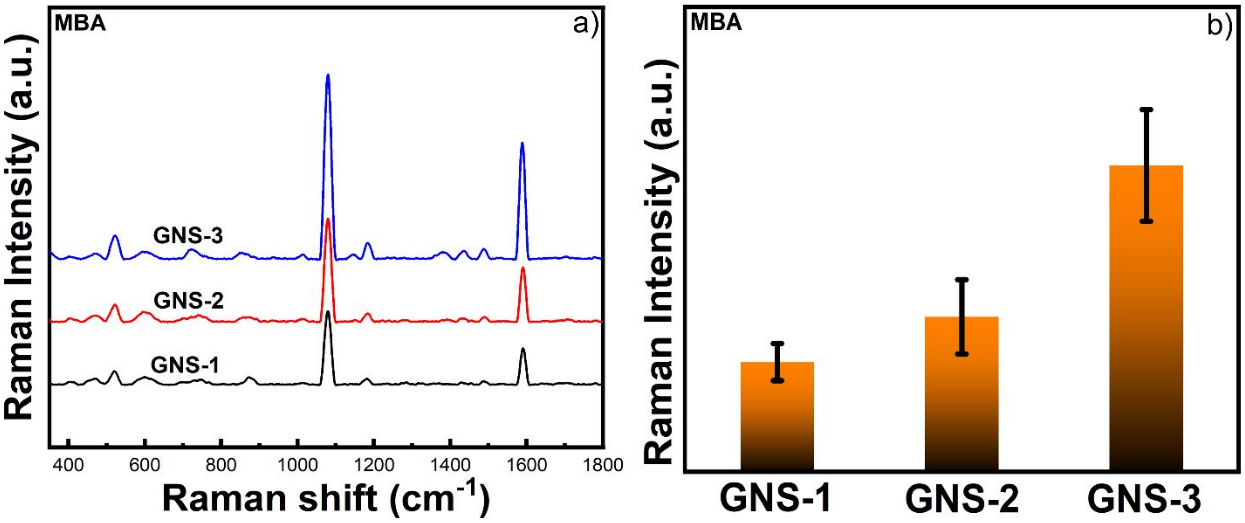

Figure S4. SERS spectra (a) and SERS intensity (b) at 1078 cm^{-1} of MBA with GNS-1, GNS-2, and GNS-3.

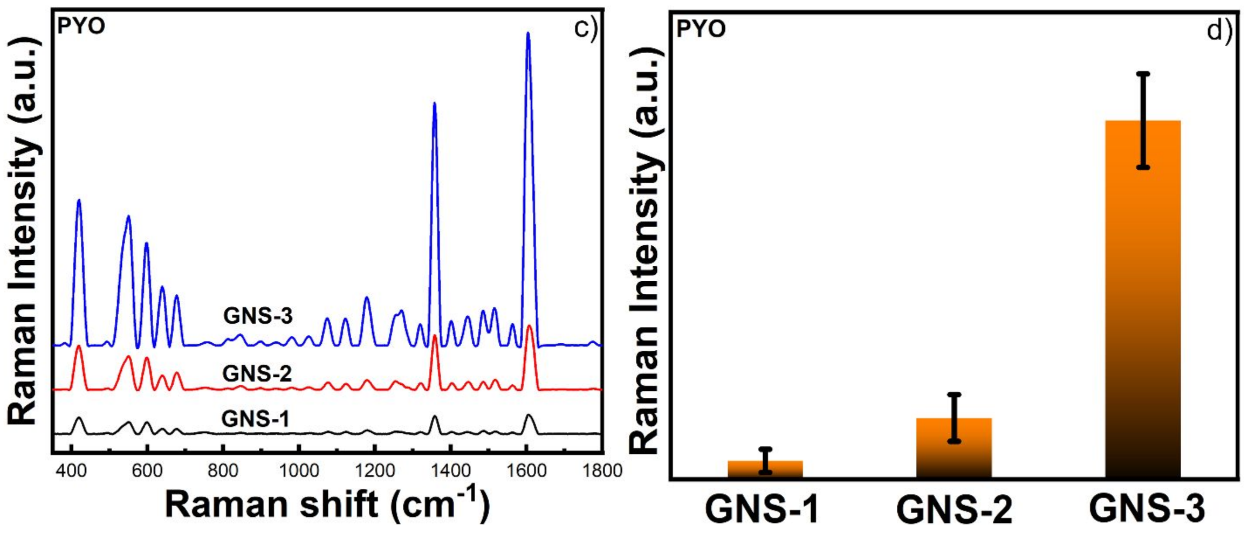

Figure S5. SERS spectra (a) and SERS intensity (b) at 1602 cm^{-1} of PYO with GNS-1, GNS-2, and GNS-3.

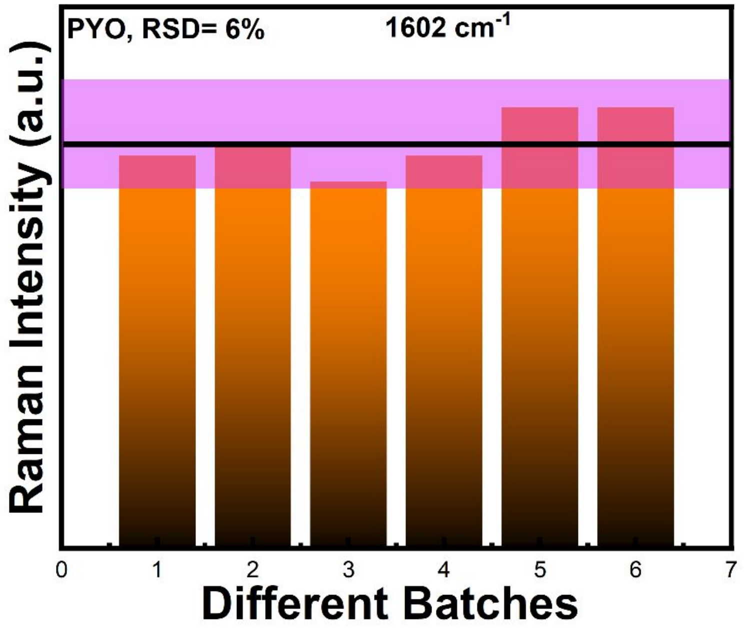

Figure S6. SERS intensity of PYO at 1602 cm^{-1} with six different batches of GNS-3.

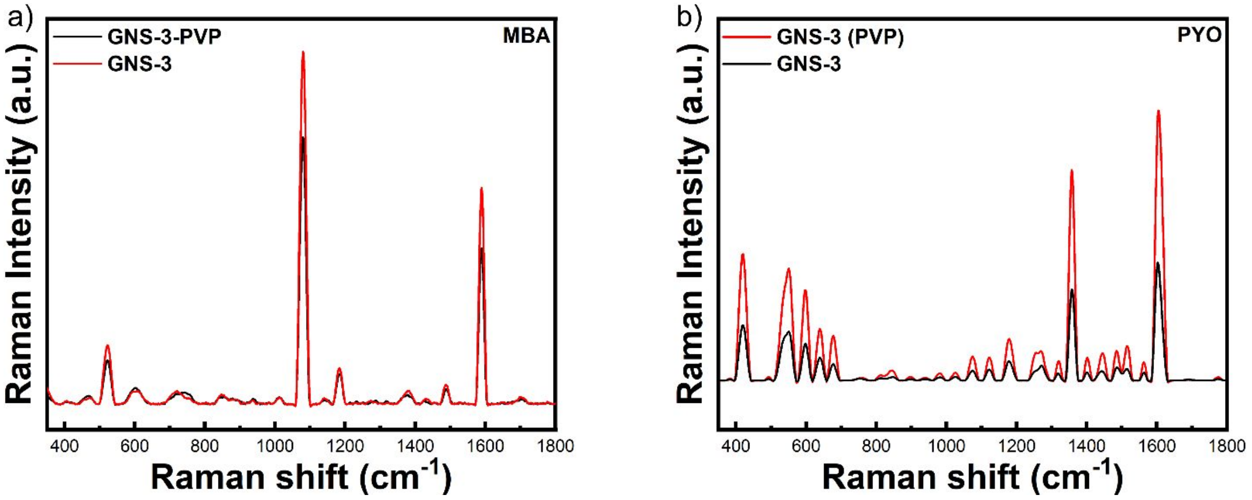

Figure S7. SERS spectra of MBA (a) and PYO (b) with PVP capped GNS-3 and surfactant-free GNS-3.

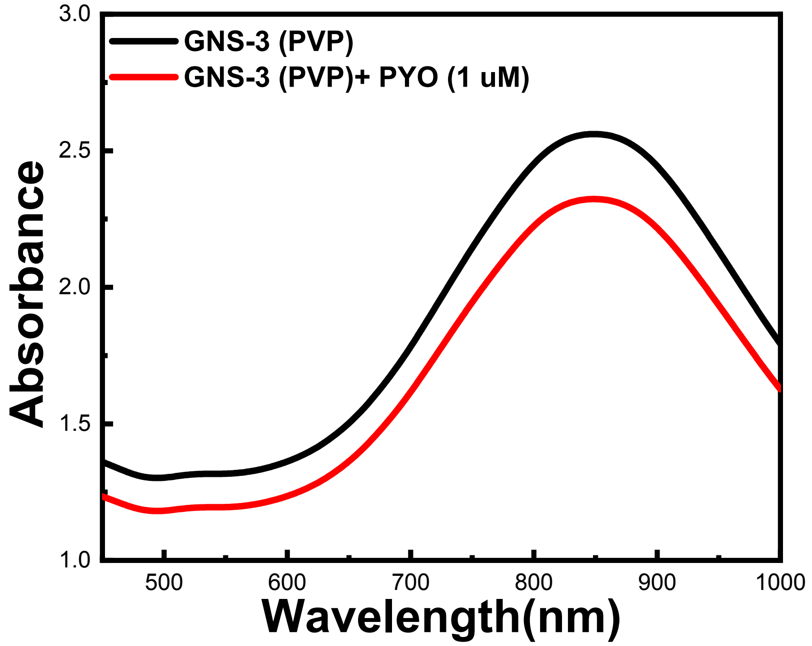

Figure S8. Absorbance spectra of PVP capped GNS-3 (black) and after 1uM PYO addition (red).

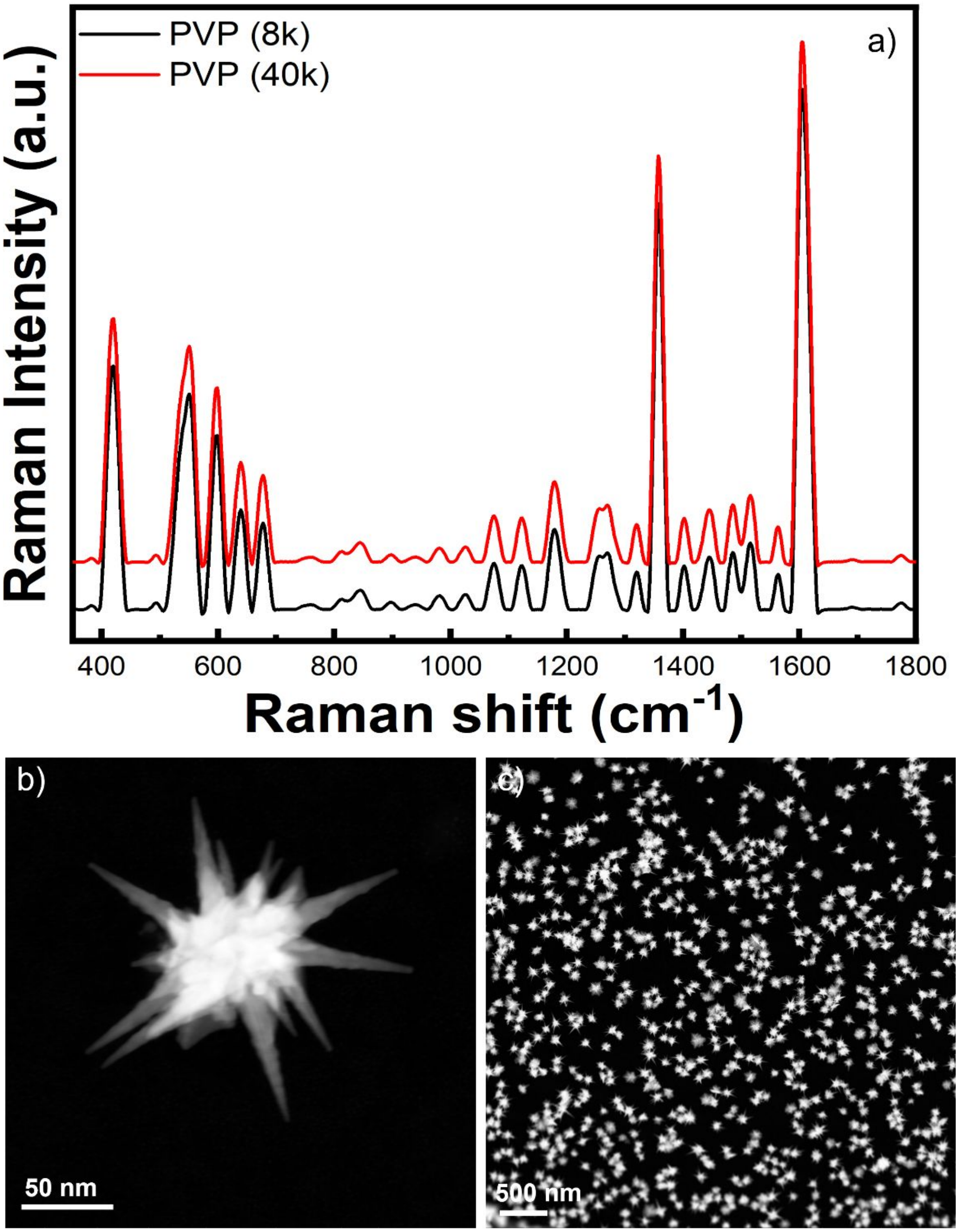

Figure S9. SERS spectra of PYO with GNS-3 coated with PVP (Mw-8k) (black) and PVP (Mw-40k) (red) (a). STEM images of PVP (40k) capped GNS-3 (b-c).

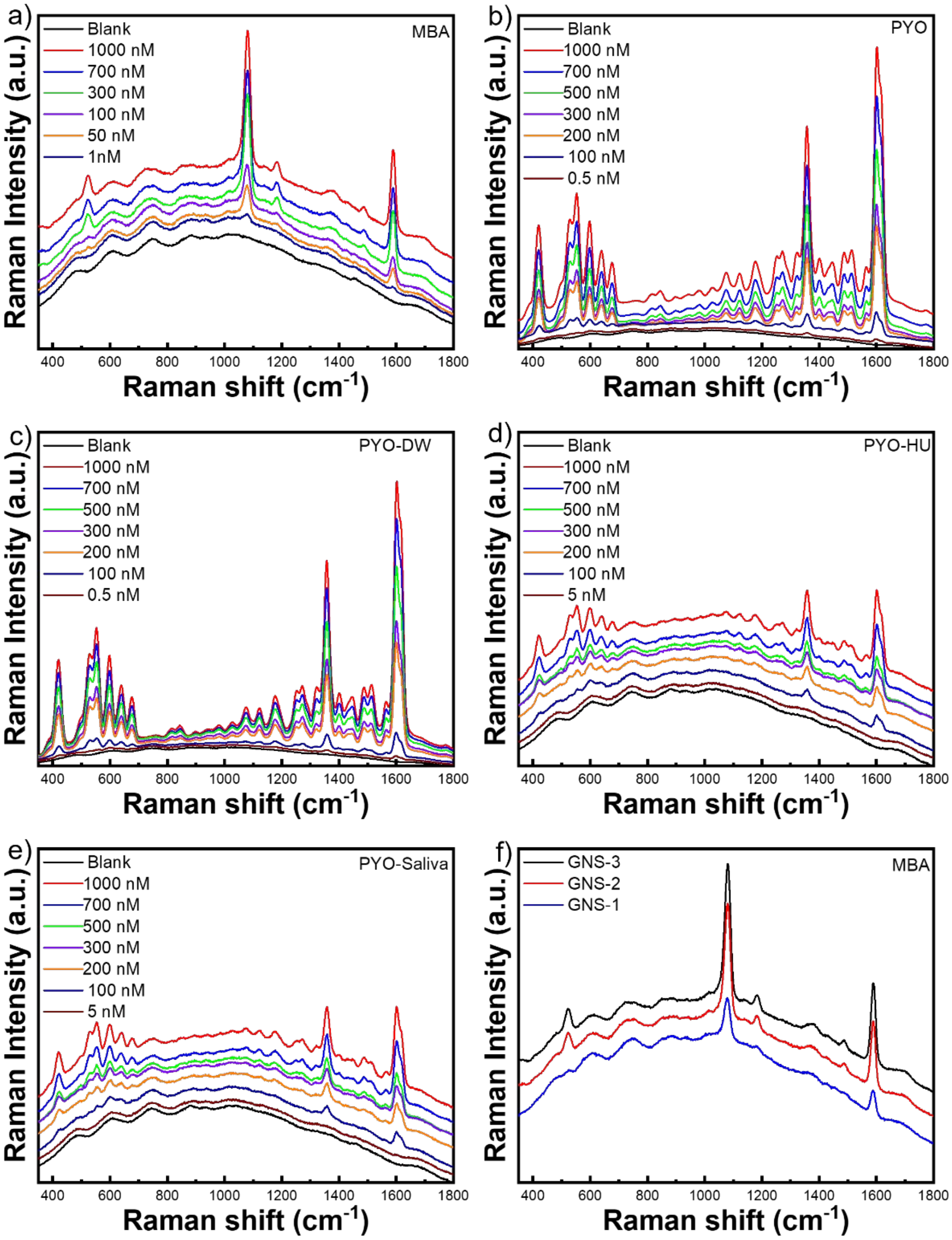
a)
Blank
1000 nM
700 nM
300 nM
100 nM
50 nM
1nM
MBA
Raman Intensity (a.u.)
Raman shift (cm-1)
b)
Blank
1000 nM
700 nM
500 nM
300 nM
200 nM
100 nM
0.5 nM
PYO
Raman Intensity (a.u.)
Raman shift (cm-1)
c)
Blank
1000 nM
700 nM
500 nM
300 nM
200 nM
100 nM
0.5 nM
PYO-DW
Raman Intensity (a.u.)
Raman shift (cm-1)
d)
Blank
1000 nM
700 nM
500 nM
300 nM
200 nM
100 nM
5 nM
PYO-HU
Raman Intensity (a.u.)
Raman shift (cm-1)
e)
Blank
1000 nM
700 nM
500 nM
300 nM
200 nM
100 nM
5 nM
PYO-Saliva
Raman Intensity (a.u.)
Raman shift (cm-1)
f)
GNS-3
GNS-2
GNS-1
MBA
Raman Intensity (a.u.)
Raman shift (cm-1)
400
600
800
1000
1200
1400
1600
1800

Figure S10. SERS spectra without background subtracted for MBA with GNS-3 (a), PYO with GNS-3 (b), PYO (drinking water) with GNS-3 (c), PYO (Human urine) with GNS-3 (d), PYO (saliva) with GNS-3, and MBA with GNS-1, GNS-2, and GNS-3.

REFERENCE

1. Atta, S.; Watcharawittayakul, T.; Vo-Dinh, T., Ultra-high SERS detection of consumable coloring agents using plasmonic gold nanostars with high aspect-ratio spikes. *Analyst* **2022,** *147* (14), 3340-3349.
2. Atta, S.; Tsoulos, T. V.; Fabris, L., Shaping Gold Nanostar Electric Fields for Surface-Enhanced Raman Spectroscopy Enhancement via Silica Coating and Selective Etching. *The Journal of Physical Chemistry C* **2016,** *120* (37), 20749-20758.
